# A common pesticide decreases foraging success and survival in honey bees: questioning the ecological relevance

**DOI:** 10.3389/fphys.2013.00037

**Published:** 2013-03-07

**Authors:** David Guez

**Affiliations:** Faculty of Science and Information Technology, School of Psychology, The University of NewcastleCallaghan, NSW, Australia

In a recent paper, Henry et al. (2012; supplemental material April 20, 2012) address the important issue of honeybee sublethal effects induced by systemic insecticides as a potential causal factor of colony collapse disorder (CCD). To evaluate mortality resulting from homing failure of foraging bees (*mhf*), Henry et al. (2012) employed radio-frequency identification (RFID) microchips to track free-ranging honey bees and then combined their *mhf* values with a colony dynamics model proposed by Khoury et al. (2011). By comparing thiamethoxam-treated and control groups they found that exposed bees were more likely to die while away from their hives. By combining the observed experimental results and model projection, the authors argue that thiamethoxam seed treatment could constitute a potential cause of CCD.

Henry et al.'s (2012) work addresses a very important area and raises some imperative issues with regards to the potential of pesticides to negatively impact upon honeybee behavior. However, its ecotoxicological and ecological significance is compromised by experimental design flaws which, if left unchallenged, could negatively impact upon future and similar ecotoxicological studies. Here I address problematic areas in the experimental design of Henry et al. (2012) and argue that whilst their work addresses a very important field of study, their conclusions are not sufficiently supported and therefore cannot be taken as ecologically relevant in this instance.

## Estimation of the daily range of thiamethoxam exposure

Henry et al. report a daily range of thiamethoxam exposure of 0.17–2.3 ng.bee^−1^.day^−1^. Henry et al. (2012) based their calculations on the method proposed by Rortais et al. (2005) assuming a thiamethoxam nectar content of 1.85 μg/kg^1^^,^^2^, and a winter oilseed rape sugar content of 10 to 30% (weight/weight) reportedly taken from Pierre et al. (1999). However, the range of thiamethoxam reported is incorrect. Although a sugar content of 30–10% (weight/weight) returns a nectar requirement of 106.7–1284.0 mg.day^−1^ (Henry et al., 2012), it actually translates into a range of exposure of 0.197–2.375 ng.bee^−1^.day^−1^. Furthermore and surprisingly, the values of sugar content reported in Pierre et al. (1999) for different oilseed varieties range from 8.25 to 66.56% and are expressed as weight/volume not as weight/weight as reported by Henry et al. (2012) suggesting that the authors have used their Pierre et al. (1999) values without conversion, overlooking the fact that the density of nectar is different from that of water, and suggest that they have arbitrarily reduced they analysis to the sugar nectar content of the “Samourai” variety (Table 3, Pierre et al., 1999).

## Is the dose used “commonly encountered”?

Henry et al. (2012) conclude their study with: “Our study clearly demonstrates that exposure of foragers to non-lethal but commonly encountered concentrations of thiamethoxam can impact forager survival, with potential contributions to collapse risk.” When considering this statement it is important to consider that winter oilseed rape flowers for around 4 weeks, and that the sugar nectar content is at its highest on the first week of flowering and at its lowest on the fourth week of flowering (Pierre et al., 1999). Henry et al.'s (2012) estimate of the range of exposure based on a nectar sugar content of 10–30% weight/weight seemingly corresponds to the field relevant data for the “Samourai” variety reported by Pierre et al. (1999) in weight/volume. In Pierre et al. (1999) the sugar content is reported as 30.08% in the first week, 19.52% in the second week, 19.06% in the third week and 10.64% in the fourth week of flowering. However in order to be commonly encountered, the dose of 1.34 ng.bee.day used by Henry et al. (2012) should be achievable four out of four weeks of flowering, regardless of Rortais' model parameters. This is particularly important since the population dynamic model output presented (Henry et al., 2012) assumes a full four weeks of exposure.

**Figure 1:**
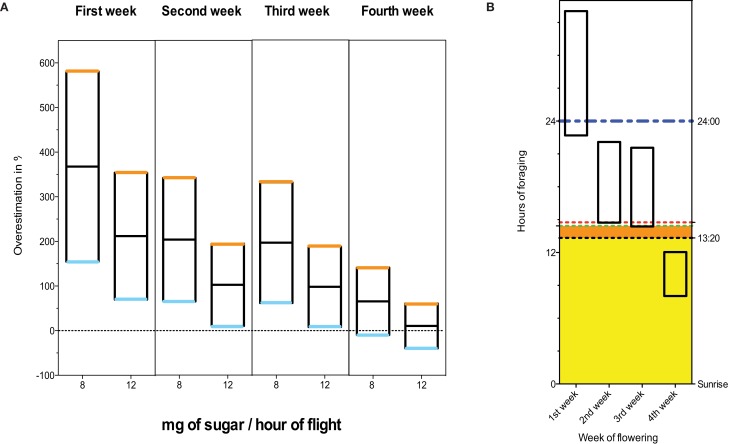
**(A)** Overestimation of the daily dose of thiamethoxam ingested by foragers. The upper boundary (orange) and lower boundary (blue) correspond to 10.7 and 4 h of flight.day^−1^ respectively. The middle bar (black) is the mean. The weekly sugar contents are taken from Table 3 in Pierre et al. (1999). All calculations are carried out as in Henry et al. (2012) [based on Rortais et al. (2005)^3^]. **(B)** Hours of foraging per day needed to reach 1.34 ng.bee^−1^ of thiamethoxam. Weekly nectar sugar contents are taken from Table 3 in Pierre et al. (1999). The upper and lower boundaries correspond to 8 and 12 mg of sugar per hour of flight. The dashed red and green lines indicate the maximum daylight in the last and third week of flowering (based on Pierre et al., 1999) at Zone Atelier *Plaine et Val de Sèvre*, French département des Deux-Sèvres (46°15'N, 0°30'W) in 2012. [Please note that the number of daylight hours would be even less for the same dates in Avignon (43°54'N, 4°52'E)^4^]. The black dashed line indicates the maximum foraging time allowed by Rortais et al. (2005) corresponding to 10 foraging bouts of 80 min each. The blue dashed line indicates 24 h.

In the absence of nectar density data it is difficult to establish with any certainty the relevance of the dose administered by (Henry et al., 2012). However, if we admit that (Pierre et al., 1999) reported the sugar content in weight/weight [As Henry et al. (2012) report], Rortais et al. (2005) proposed methods^3^ make it easy to calculate the range of possible exposure to thiamethoxam for each week of flowering, and thus to derive the difference from the actual dose used by Henry et al. (2012). Figure 1A demonstrates that the dose of 1.34 ng/bee/days is above the maximum that is predicted by Rortais et al. (2005) for 3 weeks out of 4. Even if only the fourth week of flowering was considered, the applied dose could be overestimated by as much as 140.83% (Figure 1A). It is also clear that the dose applied in this study (Henry et al., 2012) could not possibly be reached within 1 day and without foragers flying at night on the first and second week of flowering, and could barely be reached within daytime in the third week of flowering (Figure 1B). It should also be noted that these calculations assume that nectar unloading is instantaneous, and thus underestimate the time that the bee would have to spend foraging at night. Moreover, it is accepted within the field that bees rarely commence foraging immediately at sunrise. Even within the most favorable limits imposed by the model on which this study is based [namely 10 foraging trips of 80 min duration each, or 13 h 20 min of foraging (Rortais et al., 2005)], the dose administered by the Henry et al. (2012) could not be reached for 3 flowering weeks out of 4. Thus, admitting the calculation methods adopted by Henry et al. (2012), it is unlikely that free-flying bees would ever reach the daily dose of thiamethoxam that have been used by Henry et al. (2012), at least for 3 out of 4 weeks of flowering.

## Overestimation of the homing failure attributable to thiamethoxam exposure

### Acute vs. sub-chronicle

Henry et al. apply the thiamethoxam insecticide in an acute manner and claim that the dose used for oral treatment (i.e., 1.34 ng.bee^−1^) is ecologically relevant. However, this dose corresponds to what would have been absorbed by a bee in an entire day of foraging. In the context of this study, the difference between acute and sub-chronic exposure is critical. Furthermore, it is already well-established that physiological and behavioral effects vary significantly depending on whether the same dose is applied in one treatment or in many treatments over a longer period of time. For example, a human tobacco smoker inhales on average between 10.5 and 78.6 mg of nicotine per day without any immediate lethal consequences (Benowitz et al., 1984), whereas a single intake of the same amount is likely to be fatal. Gosselin et al. (1984) estimated that the acute lethal dose of nicotine is between 30 and 60 mg for a 60 kg adult (0.5 mg to 1 mg/kg). Thus, Henry et al.'s (2012) claim that the effect of an acutely applied dose of thiamethoxam is ecologically equivalent to that of a sub-chronically applied dose seems flawed.

### Improper formula

The model presented by Henry et al. (2012) is based on an incorrect formula that falsely inflates the bees' homing failure rate. Indeed, the homing failure attributed to thiamethoxam pesticide has been calculated as the following ratio of homing probabilities: (control–treatment)/control. However, since these homing probabilities are simply the ratio of returning foragers (Henry et al., 2012), it should simply be: control–treatment. Dividing by a probability (between 0 and 1), or more exactly, by the ratio of returning forager in the control group (Henry et al., 2012), results in an artificial boost to the homing failure rate which is given as an input to the model. In the case of Experiments 1 and 2 (Henry et al., 2012), this formula increases the *mhf* value by 17.5–20.4%.

## Conclusion

The ecological relevance of Henry et al.'s study is compromised by four main methodological issues. The daily range of thiamethoxam exposure is incorrectly estimated, the applied dose is uncommonly encountered, thiamethoxam is applied in an acute rather than a sub-chronical manner and the use of an incorrect formula falsely inflates the bees' homing failure rate. It is also important to acknowledge that Henry et al.'s (2012) study rest on two experimentally untested models: (1) the Rortais et al. (2005) model that proposes a direct relationship between nectar sugar content, nectar contamination and pesticide exposition (the lower the sugar content the higher the exposition), and (2) Khoury et al.'s (2011) population dynamic model. Both of these models currently lack extensive, if any, experimental validations. Based on these two points and on the highlighted shortcomings of Henry et al.'s (2012) study, the published results do not support the proposed ecological impact of thiamethoxam on honeybee mortality resulting from impaired homing fidelity. Henry et al.'s (2012) data confirms that neonicotinoid insecticides modify the behavior of honeybees as has previously been reported (e.g., Guez et al., 2001, 2003) at non-lethal, albeit ecologically unrealistic concentrations. However, more research is required to evaluate the extent to which these chemicals influence foraging behavior of honeybees operating in natural environments and whether prolonged exposure to neonicotinic compounds might contribute to a multifactorial etiology of CCD.

## Footnotes

^1^Afssa–dossier n° 2009-1235 – CRUISER 350, http://facm.viabloga.com/files/DIVE2009ha1235 AMM Cruiser.pdf

^2^Anses file n°2007-3336- CRUISER OSR, http://www.anses.fr/Documents/DPR2007ha3336.pdf

^3^Range estimations are based on the estimated energetic demand of a forager per hour of flight [8–12 mg of sugar per hour of flight (Rortais et al., 2005)], an average number of foraging bouts of 10 per forager and per day, with a duration ranging from 30 to 80 min per foraging bouts and assuming that 80% of the length of a foraging bout is spent flying (Rortais et al., 2005). Foragers are thus expected to fly on average 4–10.7 h/day of foraging and thus need to absorb 32–128.4 mg of sugar per day. If we assume, as Rortais et al. (2005) that contaminated nectar is the only available source of sugar, we can calculate the daily dose of thiamethoxam using the equation given below (the units are in subscript)

$${\left[\text{Dose of thiamethoxam}\right]}_{\text{ng}.{\text{bee}}^{\text{-1}}.{\text{day}}^{\text{-1}}}=\frac{{\left[\text{Thiamethoxam}\right]}_{\mu \text{g}.{\text{kg}}^{\text{-1}}}}{10\times {\left[\text{Nectar sugar content}\right]}_{\text{g/100g}}}\times {\left[\text{Daily sugar need}\right]}_{\text{mg}.{\text{bee}}^{\text{-1}}.{\text{day}}^{\text{-1}}}.$$

^4^Daylight duration for both location can be obtained from http://www.esrl.noaa.gov/gmd/grad/solcalc/
